# Tumor control by human cytomegalovirus in a murine model of hepatocellular carcinoma

**DOI:** 10.1038/mto.2016.12

**Published:** 2016-04-27

**Authors:** Amit Kumar, Laurie Coquard, Sébastien Pasquereau, Laetitia Russo, Séverine Valmary-Degano, Christophe Borg, Pierre Pothier, Georges Herbein

**Affiliations:** 1 Department of Virology, Pathogens & Inflammation Laboratory, University of Franche-Comté and COMUE Bourgogne Franche-Comté University, UPRES EA4266, SFR FED 4234, CHRU Besançon, Besançon, France; 2 Department of Pathology, CHRU Besançon, Besançon, France; 3 Department of Medical Oncology, INSERM UMR1098, EFS Bourgogne Franche-Comté, Besançon, France; 4 Department of Virology, Pathogens & Inflammation Laboratory, UPRES EA4266, SFR FED 4234, CHU Dijon, Dijon, France

## Abstract

Although viruses can cause cancer, other studies reported the regression of human tumors upon viral infections. We investigated the cytoreductive potential of human cytomegalovirus (HCMV) in a murine model of human hepatocellular carcinoma (HCC) in severe-immunodeficient mice. Infection of HepG2 cells with HCMV resulted in the absence of tumor or in a limited tumor growth following injection of cells subcutaneously. By contrast all mice injected with uninfected HepG2 cells and with HepG2 cells infected with UV-treated HCMV did develop tumors without any significant restriction. Analysis of tumors indicated that in mice injected with HCMV-infected-HepG2 cells, but not in controls, a restricted cellular proliferation was observed parallel to a limited activation of the STAT3-cyclin D1 axis, decreased formation of colonies in soft agar, and activation of the intrinsic apoptotic pathway. We conclude that HCMV can provide antitumoral effects in a murine model of HCC which requires replicative virus at some stages that results in limitation of tumor cell proliferation and enhanced apoptosis mediated through the intrinsic caspase pathway.

## Introduction

Human cytomegalovirus (HCMV) is an opportunistic, species-specific herpesvirus that infects a large proportion of the population worldwide and often results in an asymptomatic latent infection in healthy individuals. However, in immunosuppressed patients HCMV infection results in significant mortality and morbidity.^1,2^ During the last decade, using highly sensitive techniques, several groups have detected the presence of HCMV in a large proportion of glioma, colon cancers, breast cancers, prostate cancers, skin cancers, salivary gland cancers, and medulloblastomas.^3–10^ In addition, several groups fail to detect the HCMV signatures in several kinds of human-derived tumors making relationship between HCMV and cancer highly controversial and dynamic.^7,11,12^ In a recent large-scale multiethnic case–control study, higher levels of antibody against HCMV have been reported in healthy controls than in breast cancer patients, suggesting a link between HCMV and breast cancer.^13^ Moreover, HCMV could act as an “oncomodulator” both on the tumor cells and the tissue microenvironment to promote inflammation, cell cycle progression, immune escape, tumor spreading, angiogenesis, and reduced survival.^14–16^ By contrast a stage-specific beneficial role for HCMV infection has been reported in adult T-cell leukemia/lymphoma^17,18^ and a reduction in early relapse risk was recently observed after allogeneic hematopoietic cell transplantation associated with HCMV reactivation.^19–21^ Additionally, HCMV has shown tumor control in a model of bone marrow transplantation and acute liver-infiltrating B-cell lymphoma.^22,23^ Besides HCMV, live-attenuated measles virus and attenuated vaccinia virus induce regression of human breast tumor xenografts and human lymphoma xenografts in immunodeficient mice, respectively.^24,25^ Recently, remission of disseminated cancer was obtained after systemic oncolytic virotherapy using a recombinant oncolytic measles virus derived from an attenuated measles virus strain.^26,27^ In addition, combination of epigenetic modifiers such as resminostat and an oncolytic measles vaccine virus showed effective killing of human hepatocellular carcinoma (HCC) *in vitro*.^28^

Here, we investigated the cytoreductive potential of HCMV in a murine model of human hepatocellular carcinoma in severe immunodeficient mice and showed that the HCMV infection delayed the development of HCC tumors in nude mice due to both reduced proliferation and increased caspase-dependent apoptosis of tumoral cells.

## Results

### HCMV infection of HepG2 cells inhibits tumor growth in xenografted mice

**Figure 1:**
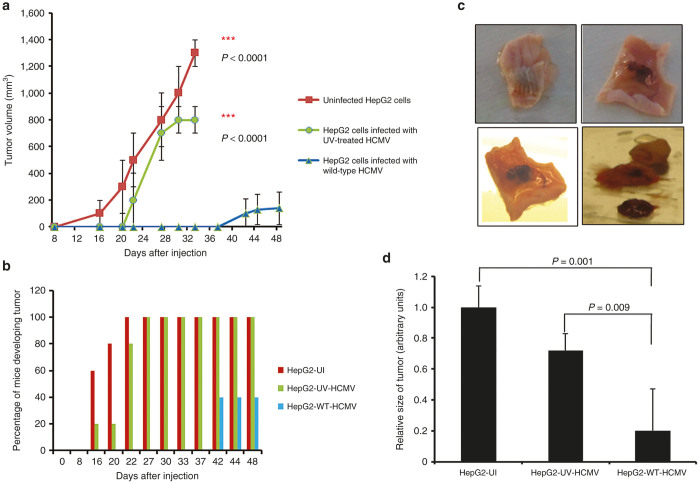
Effect of HCMV on tumor growth of HepG2 cells in xenografted mice. (**a**) HepG2 cells were either left uninfected or infected with UV-inactivated HCMV (UV-HCMV) or wild-type HCMV (AD169). After 24 hours of infection, nude balb/c mice were injected subcutaneously with HepG2 cells (uninfected: HepG2-UI, infected with UV-inactivated HCMV: HepG2-UV-HCMV, and wild-type HCMV: HepG2-WT-HCMV). Tumor growth was assessed as shown in figure and tumor volume was determined as described in Materials and Methods. (**b**) Percentage of mice developing tumor in each group (HepG2-UI; *n* = 5, HepG2-UV-HCMV; *n* = 5, HepG2-WT-HCMV; *n* = 5) is shown. (**c**) Representative picture of tumor retrieved from mice injected with HepG2 cells infected with wild-type HCMV. (**d**) Size of tumor was recorded as described in Materials and Methods. Graph represents the relative size of tumor in each group with respect to control group (HepG2-UI).

Previously, we observed the increased number of colonies in soft agar seeded with HepG2 cells infected with laboratory HCMV strain AD169 after 1 day of infection as compared with uninfected cells or cells infected with UV-treated HCMV.^29^ Thus, we hypothesized that tumor formation should be also increased *in vivo*. To test this hypothesis, we infected HepG2 cells with HCMV strain AD169 at 1 multiplicity of infection (MOI). After 24 hours of infection, 5 × 10^6^ cells were subcutaneously injected to Balb/c nude mice, usually a recognized experimental model for human xenograft.^30^ As controls, mice were also injected with HepG2 cells infected with UV-inactivated HCMV strain AD169 or with uninfected HepG2 cells. Tumor volume was assessed *in vivo* at different time intervals postinjection up to day 48 (Figure 1a). Surprisingly, we observed that tumor growth was delayed in the wild-type HCMV-infected HepG2 cells group (HepG2-WT-HCMV) compared with the controls (Figure 1a). Mice injected with uninfected HepG2 cells (HepG2-UI) and with HepG2 cells infected with UV-inactivated HCMV (HepG2-UV-HCMV) began to develop tumors on day 16 (Figure 1a,b). By contrast, mice injected with HepG2 cells infected with wild-type HCMV began to develop tumors only on day 42 postinjection (Figure 1a–c). Additionally, out of five mice injected with HepG2 cells infected with HCMV, only two (40%) developed tumors (Figure 1b) and the tumor size was even smaller (*P* = 0.001, HepG2-UI versus HepG2-WT-HCMV) (Figure 1d).

**Figure 2:**
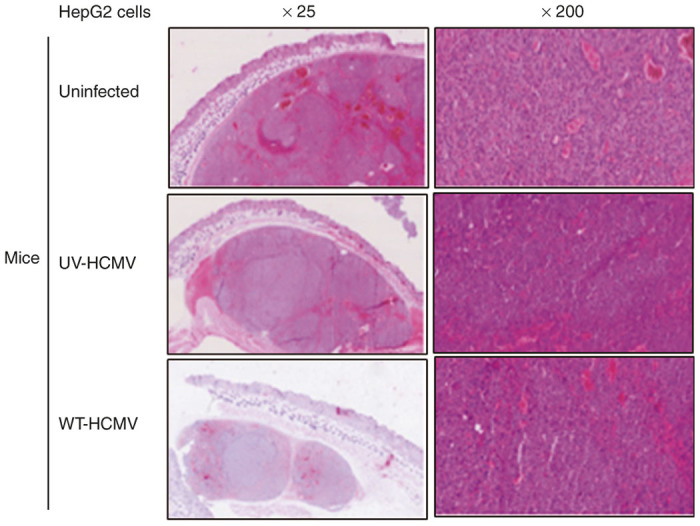
Morphological assessment of tumors retrieved from different mice groups using immunohistochemistry (IHC). Upon killing the mice, tumors and organs (liver and lung) were retrieved and half of the tissues were fixed in formalin and half were stored in ice-cold PBS for molecular analysis. Sections were made and initial assessment of the tissue was made by IHC using hematoxylin/eosin (H/E) staining as mentioned in Materials and Methods. Figure is a representative picture of tumor sections stained with H/E retrieved from each mice group (original magnification ×25, ×200).

To investigate the mechanisms involved in tumor growth restriction, we collected subcutaneous tumors and organs (liver and lung) from xenografted mice. We did not find any tumoral tissue in the liver or the lung (data not shown), indicating the absence of metastases in our murine model of xenografts. The morphological features of the tumors were assessed using hematoxylin and eosin staining (Figure 2). We observed that the tumors were nodular, well delimited, and nonencapsulated. Architecture was massive, nodular and pseudoglandular in rosette. Cells were enlarged with abundant eosinophilic cytoplasm. Nuclei were voluminous, clear, round, with high number of nucleolus including a central one. Nucelocytoplasmic ratio was increased. Mitoses were numerous (40 mitoses per 10 fields, at magnification factor ×40). Vascularization was rich with haemorrhages. No significant morphological difference was found in the tumor biopsies of the different groups (HepG2-UI, HepG2-UV-HCMV, and HepG2-WT-HCMV) (Figure 2).

### Limited HCMV replication and restricted tumor growth

**Figure 3:**
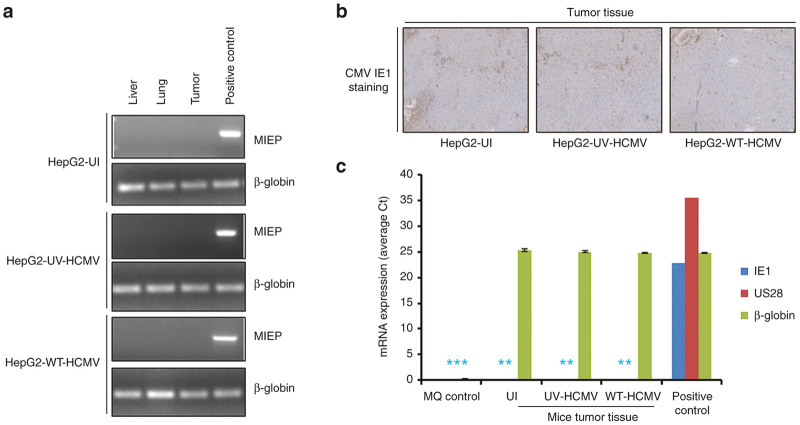
Absence of HCMV signatures (DNA, transcripts, and proteins) in tissues retrieved from different mice groups (HepG2-UI, HepG2-UV-HCMV, and HepG2-WT-HCMV). (**a**) Screening for the presence of HCMV DNA in organs (liver and lung) and tumors retrieved from different mice groups using polymerase chain reaction. DNA was isolated from tumors and organs isolated from different mice groups. Equal amount of DNA was amplified using MIEP and β-globin primers. Amplicons were electrophoresed on 2 % agarose gel and stained with Sybr green I nucleic acid stain. Positive control is viral DNA isolated from the supernatants of MRC5 post 3 days of infection. (**b**) Representative pictures of tumor biopsies from different mice group stained from HCMV IE1-Ag using IHC. No specific CMV IE staining was detected. (**c**) Absence of HCMV IE1 and US28 transcripts in tumors retrieved from mice infected with WT-HCMV. RNA was isolated from tumors obtained from mice. Equal amount of RNA was reverse transcribed into cDNA using oligo dT primer and presence of IE1, US28, and β-globin was determined using real-time PCR. Positive control is RNA isolated from MRC5 infected with AD169 post 3 days of infection. * denotes that no Ct value was observed, small bars have been added for presentation purpose only. MIEP, major immediate early promoter.

We observed that restriction of tumor formation depends on infection of HepG2 cells with a replicative virus, since injection of HepG2 cells infected with UV-inactivated HCMV (HepG2-UV-HCMV) did not significantly delay tumor formation (Figure 1a,b). Therefore, we assessed the presence of HCMV DNA in tumor samples, but also in lung and liver samples, isolated from the three mice groups at days 33 and 48 postinjection. We determined the presence of major immediate early promoter DNA by semiquantitative PCR (Figure 3a, Supplementary Table S1 and Supplementary References). We did not find the presence of HCMV DNA in the biopsies tested (Figure 3a). Additionally, a real-time PCR for HCMV quantification was performed, and also did not allow the detection of viral DNA neither in the tumors nor in lung and liver biopsies isolated from mice injected with HepG2 cells infected with wild-type HCMV (data not shown). In agreement with the absence of viral DNA in tumor biopsies, we did not detect IE1 HCMV antigen in tumor biopsies from either mice using immunohistochemistry (IHC) staining (Figure 3b). Finally, since HCMV early proteins such as US28 have been reported to induce apoptosis and thereby could restrict tumor growth,^31^ we assessed the expression of US28 and IE1 transcripts by quantitative real-time PCR using relative quantitation method (Figure 3c). We did not detect US28 and IE1 transcripts neither in tumor tissue from mice injected subcutaneously with HCMV-infected HepG2 cells nor from the control groups using RT-PCR (Figure 3c).

### HCMV infection limits HepG2-cell proliferation *in vivo.*

**Figure 4:**
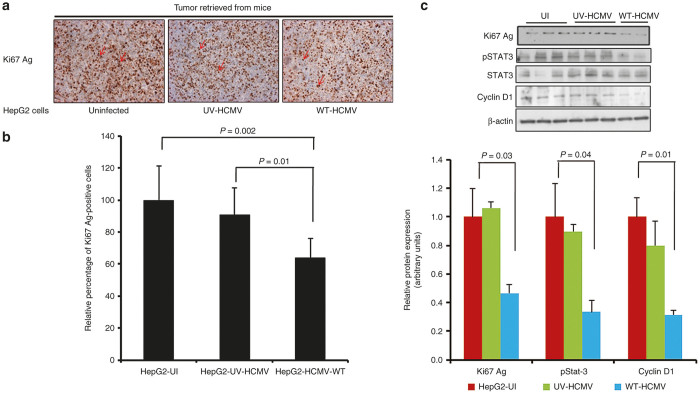
Tumor retrieved from mice injected with HepG2 infected with wild-type HCMV exhibits low proliferation and limited activation of STAT3-cyclin D1 axis. Ki67 antigen is considered as one of the hallmarks of cell proliferation. IHC was performed to determine the Ki67 Ag labeling index in tumor tissues retrieved from the different mice group. (**a**) Representative picture of tumor biopsies retrieved from mice showing Ki67 Ag labeling (brown precipitate shown by red arrow) (original magnification ×200). (**b**) Percentage of cells positive for Ki67 Ag were determined *in silico* by counting positive cells using automated software and plotted as a graph (*P* = 0.002; UI versus WT-HCMV, *P* = 0.01; UV-HCMV versus WT-HCMV). (**c**) Expression of Ki67 Ag, phosphoStat-3, total Stat-3, cyclin D1, and β-actin was determined by western blotting. Tumor tissues were weighed and crushed in liquid nitrogen. Protein was extracted using RIPA buffer and quantitated using Bradford protein estimation method and expression of proteins was determined using western blotting as described in Materials and Methods. Histogram represents Ki67 Ag, phosphoSTAT3, and cyclin D1 expression as measured using image J software. WT-HCMV, wild-type HCMV

We measured the expression of the nuclear antigen Ki67 (Ki67 Ag), a hallmark of cell proliferation, using IHC staining (Figure 4a,b) and western blotting (Figure 4c). Ki67 Ag labelling was homogeneous with ~15–20% of cells stained in tumor biopsies collected from mice-injected HepG2 cells infected with HCMV. In comparison, the positivity for Ki67 Ag in the control groups was significantly higher with a mean of 30% (*P* = 0.002, HepG2-UI versus HepG2-HCMV-WT; *P* = 0.01, HepG2-UV-HCMV versus HepG2-HCMV-WT) (Figure 4a,b). In agreement with the IHC results, tumor tissue derived from HepG2-HCMV-WT exhibits lower levels of Ki67 Ag as compared with HepG2-UV-HCMV and HepG2-UI as shown by western blotting (Figure 4c).

Cyclin D1 is a regulator of cyclin-dependant kinases which is involved in cell cycle progression and proliferation and is overexpressed in several types of cancers including breast cancer and HCC.^32^ We assessed the level of cyclin D1 protein in tumor biopsies from mice from different group (HepG2-UI, HepG2-UV-HCMV, and HepG2-WT-HCMV). We observed a decreased expression of cyclin D1 in the tumors of mice injected with HepG2 cells infected with HCMV compared with the controls (uninfected HepG2 cells and HepG2 cells infected with UV-treated HCMV), using western blotting (Figure 4c). Since cyclin D1 over expression in HCC is mediated by the IL-6-STAT3 axis,^33^ we also determined the expression of phosphoSTAT3 in tumor tissue among different groups. We observed decreased STAT3 activation (lower level of STAT3 phosphorylation) in tumors of mice injected with HepG2 cells infected with HCMV compared with the controls (uninfected HepG2 cells and HepG2 cells infected with UV-treated HCMV), using western blot analysis (Figure 4c). Altogether, our data indicate decreased activation of the STAT3-cyclin D1 axis in tumors of mice injected with HepG2 cells infected with HCMV compared with the controls.

### HCMV infection reduces the tumor-formation potential of HepG2 cells

**Figure 5:**
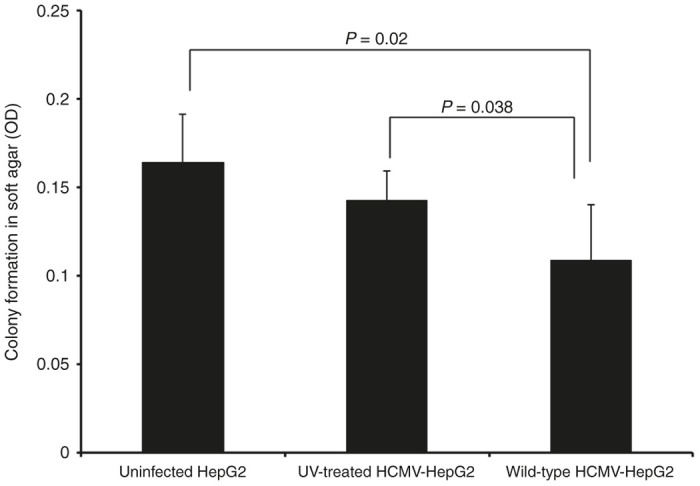
Tumor cells retrieved from mice infected with WT-HCMV showed low tumorogenic potential. One fourth of the tumor tissues isolated from different mice groups were stored in 1× ice-cold PBS for determination of tumorogenic potential using soft agar assay. Individual populations of the cells were obtained from tumor tissues as described in the Materials and Methods. Cells were seeded in soft agar assay and post 8 days of seeding colony formation was determined using MTT assay. *P* = 0.02 (HepG2-UI versus HepG2-WT-HCMV); *P* = 0.038 (HepG2-UV-HCMV versus HepG2-WT-HCMV). HCMV, human cytomegalovirus; HepG2-UI, uninfected HepG2 cells; HepG2-UV-HCMV, UV-inactivated HCMV; HepG2-WT-HCMV, wild-type HCMV-infected HepG2 cells group; WT-HCMV, wild-type HCMV.

Due to decreased proliferation and lower activation of the STAT3-cyclin D1 axis in HepG2 cells following exposure to HCMV in tumor biopsies, we assessed whether HepG2 cells infected with HCMV have a reduced oncogenic potential. Therefore, we isolated the cells from the tumor tissue retrieved from mice under sterile condition and seeded the cells for soft agar colony formation assay, which is one of the most stringent assays for detecting the malignant transformation of cells, to directly test whether tumor-derived HepG2 cells infected initially with wild-type HCMV were less transformed compared with uninfected HepG2 cells or HepG2 cells infected initially with UV-treated HCMV. After 8 days of culture, we observed decreased formation of colonies in soft agar that had been seeded with tumor cells derived from mice injected with HepG2 cells infected with HCMV compared with controls (Figure 5). In tumor biopsies from mice injected with HepG2 cells infected with HCMV, a lower number of colonies was observed compared with biopsies from mice injected with uninfected HepG2 cells (*P* = 0.02) or with HepG2 cells infected with UV-inactivated virus (*P* = 0.038) (Figure 5). These results indicate that the *in vivo* cellular transformation potential of HepG2 cells was hampered following *in vitro* infection with wild-type HCMV.

### HCMV induces caspase-dependent apoptosis of HepG2 cell tumors in a p53-independent manner.

**Figure 6:**
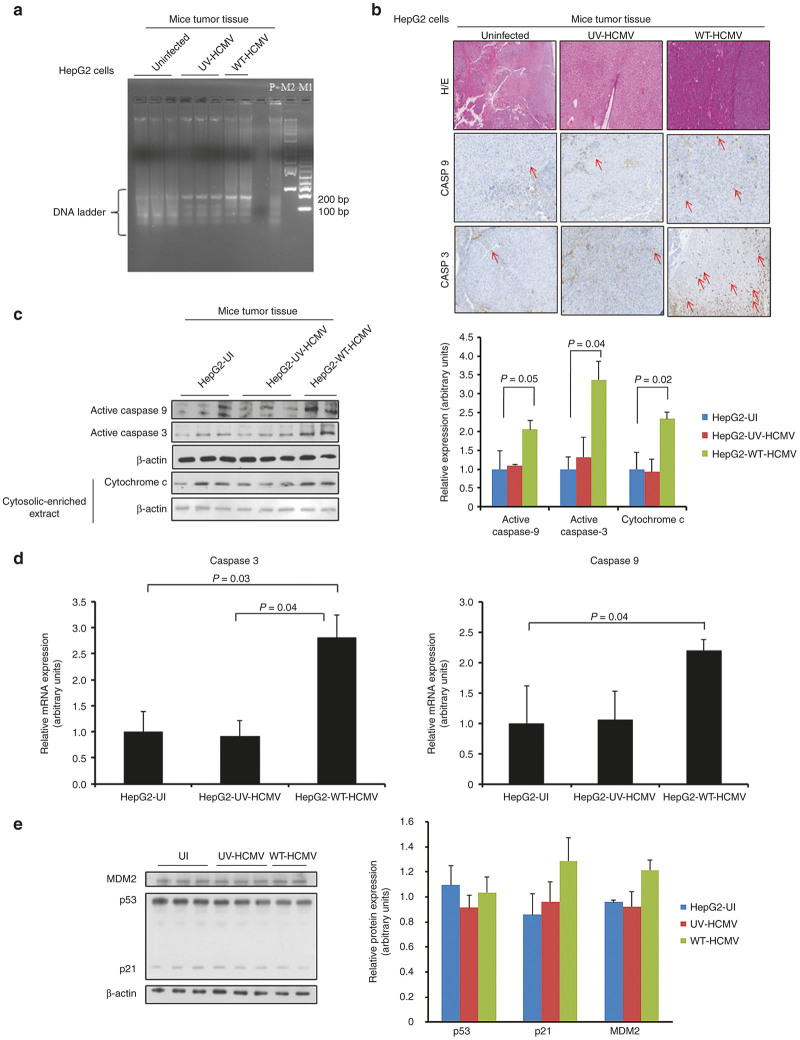
HCMV induces caspase-mediated apoptosis in hepatocellular carcinoma cells in a p53-independent manner. (**a**) To have a quick glimpse in the extent of apoptosis in tissue isolated from different groups (HepG2-UI, HepG2-UV-HCMV, and HepG2-WT-HCMV), genomic DNA was isolated from tumor tissues. DNA was visualized on 2% agarose gel stained with Sybr green I nucleic acid stains. M1 is a 100-bp DNA ladder (Fermentas, generuler 100-bp DNA ladder cat # SM0243) and M2 is a 1-kb DNA ladder (Fermentas, generuler 1-kb DNA ladder cat # SM0313). P: positive control is the genomic DNA isolated from HepG2 cells treated with Akt1/2 inhibitor for 30 hours in serum-deprived condition. (**b,c**) Further to understand the mechanism of delayed appearance of tumor in mice injected with WT-HCMV infected cells, expression of caspase-3 and caspase-9 proteins and transcripts in tumor biopsies was determined using immunohistochemistry (**b**), western blotting (**c**) and real-time quantitative PCR (**d**). For cytochrome c detection in the cytosol, cytosolic-enriched tumor fraction was prepared as described.^59^ As a loading control, levels of β-actin protein were determined. Mice injected with WT-HCMV-infected HepG2 cells exhibited higher levels of caspase-3 and caspase-9 in tumor biopsies as shown by IHC (**b**) and western blotting (**b**). (**d**) Relative expression of caspase-3 and caspase-9 transcripts was determined by reverse-transcriptase real- time PCR (RT-PCR) using 2^−ΔΔCT^ method.^58^ (**e**) No significant difference was found in the expression of p53, p21, and MDM2 in tumor tissues isolated from different groups (HepG2-UI, *n* = 3; HepG2-UV-HCMV, *n* = 3; and HepG2-WT-HCMV, *n* = 2). Protein was isolated from tumors retrieved from different mice groups and expression of p53, p21 and MDM2, and β-actin was determined by western blotting. HCMV, human cytomegalovirus; HepG2-UI, uninfected HepG2 cells; HepG2-UV-HCMV, UV-inactivated HCMV; HepG2-WT-HCMV, wild-type HCMV-infected HepG2 cells group; WT-HCMV, wild-type HCMV.

Since we observed restriction of tumor formation in mice injected with HepG2 cells infected with HCMV, we assessed whether apoptosis could be involved. Using the detection of DNA fragmentation as a marker of apoptosis (apoptotic DNA ladder), we observed increased apoptosis in tumor biopsies from mice injected subcutaneously with HCMV-infected HepG2 cells compared with the control groups (uninfected HepG2 cells and HepG2 cells infected with UV-treated HCMV) (Figure 6a). Since cytomegalovirus has already been reported to induce apoptosis in several experimental models,^31,34–36^ we performed IHC staining to detect the expression of caspase-9 and caspase-3 on tissue biopsies. We observed that several HepG2 cells were positive for both caspase-9 and caspase-3 in tumor biopsies from mice injected subcutaneously with HCMV-infected HepG2 cells (Figure 6b, middle right and lower right panels). The control groups (uninfected HepG2 cells and HepG2 cells infected with UV-treated HCMV) did not show any significant caspase-3 and caspase-9 staining (Figure 6b). In agreement with the IHC results, we observed increased expression of caspase-9 and caspase-3 in tumor tissue from mice injected subcutaneously with HCMV-infected HepG2 cells but not from the control groups using western blotting (Figure 6c). Since our results are in agreement with the induction of the intrinsic apoptotic pathway in tumor biopsies from mice injected subcutaneously with HCMV-infected HepG2 cells, we assessed cytochrome c-induced caspase-9 activation in our xenografted murine model. We observed increased expression of cytochrome c in the cytosolic-enriched preparation of tumor tissues isolated from HepG2-WT-HCMV group as compared with other control groups (Figure 6c). In agreement with the role of caspase-mediated apoptosis in tumor biopsies, we observed increased expression of caspase-9 and caspase-3 transcripts in tumor tissue from mice injected subcutaneously with HCMV-infected HepG2 cells but not from the control groups using RT-PCR assay (Figure 6d). To further assess the involvement of the extrinsic apoptotic pathway, we determine the relative abundance of transcripts of some key players (TNFR1, TNFR2, Fas, Fas ligand (Fas-L), and caspase 8) using real-time PCR by relative-quantitation method. We did not find any significant difference in the levels of these transcripts among different groups (see Supplementary Figure S1)

**Figure 7:**
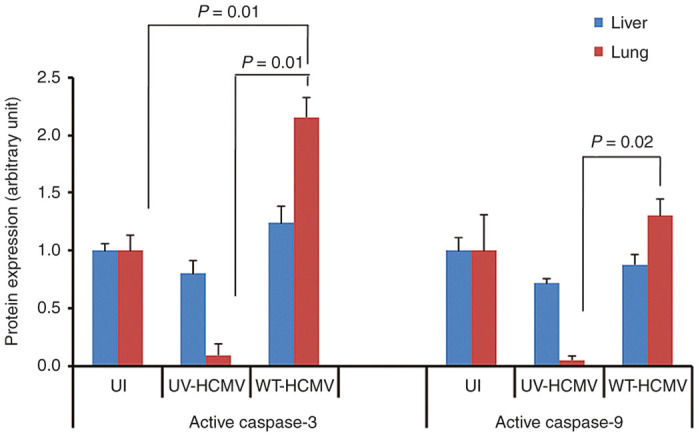
Caspase activation in lung tissue of xenografted mice injected with HepG2 cells infected with WT-HCMV. Levels of active caspase-3 and active caspase-9 were determined in the organs (liver and lung) retrieved from different mice groups using western blotting. Intensity of bands was quantitated using image J software and plotted as a graph. HCMV, human cytomegalovirus; WT-HCMV, wild-type HCMV.

Viral-induced apoptosis can be p53 dependent^37^ or p53 independent.^38^ To further characterize the nature of apoptosis in different tumor tissues retrieved from different mice groups, we determined the expression of p53, p21, and MDM2 using western blotting (Figure 6e). We did not find any significant change in the expression of these molecules, suggesting that HCMV induces apoptosis in our murine model in a p53-independent manner (Figure 6e). Finally, we assessed apoptosis at distance of tumor as already reported for murine CMV infection.^22^ We found caspase-3 and caspase-9 activations especially in lung tissue of mice injected subcutaneously with HCMV-infected HepG2 cells but not from the control groups using western blotting (Figure 7).

## Discussion

In this study, we observed that infection of HepG2 cells with HCMV delayed the tumor growth in xenografted mice. Further investigations of the mechanisms involved revealed a restriction in both HepG2 cell proliferation and tumorogenicity. The delayed tumor growth of engrafted HCMV-infected HepG2 cells resulted from decreased cell proliferation and induced apoptosis via an intrinsic caspase-dependent pathway.

Our previous *in vitro* study indicated an oncomodulator role of HCMV in the HepG2 cell line.^29^ To further assess the role of HCMV infection of HepG2 cells *in vivo*, we used a murine model of engrafted HepG2 cells infected *in vitro* with HCMV. Since HCMV is a strictly species-specific pathogen, animal models for HCMV study are limited. Several approaches have been employed in overcoming the issue of HCMV cross-species barrier. The implantation of humanized tissue such as liver/fetal thymus or retinal tissue followed by infection with HCMV has been successfully tried in the past.^39–41^ Another more convenient way is to infect human cells *in vitro* and then implant in mice using matrigel or other supports such as gelfoam.^42^ We used latter strategy for our study (Figure 1). We injected five millions of HepG2 cells either uninfected or infected with wild-type HCMV or UV-inactivated HCMV in the Balb/c nude mice. We observed the tumor formation in all mice injected with uninfected HepG2 cells and HepG2 cells infected with UV-inactivated HCMV but only in a limited number of mice injected with HepG2 cells infected *in vitro* with wild-type HCMV. Whereas in the former groups all mice exhibited tumors, in the latter group only 40% of mice showed tumor formation with delayed tumor appearance, usually on day 42 postinjection (Figure 1a,b). Results suggest that presence of replicating wild-type virus was prerequisite for the absence of tumor and/or delayed tumor formation. Upon killing mice, we assessed the presence of HCMV in mice tissue (tumor, liver, and lung) using conventional and real-time PCR (Figure 3a). We did not detect HCMV genome in either tissue. This observation is in agreement with previous studies where level of HCMV became undetectable after few weeks of HCMV infection in a murine model.^39^ Our results are contradictory to another study which describes increased size of tumors in mice implanted with neurospheres infected with HCMV compared with mice injected with mock-infected neurospheres.^43^ The difference in cell types used, HCMV strains, and mice strains employed in these study may contribute to the different results.

To understand the mechanism(s) of delayed tumor formation in mice injected with HepG2 cells treated with the wild-type HCMV, we performed hematoxylin/eosin staining (Figure 2) and IHC (Figure 4a). We found decreased cellular proliferation as detected by lower expression of Ki67Ag in tumor biopsies from HepG2-WT-HCMV-injected mice as compared with controls (*P* = 0.002, HepG2-UI versus HepG2-WT-HCMV mice; *P* = 0.01, HepG2-UV-HCMV versus HepG2-WT-HCMV mice) (Figure 4a,b). We also observed decreased STAT3 activation and cyclin D1 expression in tumor biopsies from HepG2-WT-HCMV-injected mice as compared with controls (Figure 4c). This result is in agreement with the previously reported decreased STAT3 and cyclin D1 activation at day 6 following acute infection of HepG2 cell cultures with HCMV.^29^ Since STAT3 activation and cyclin D1 have been reported to be critical for hepatocyte transformation and HCC appearance,^44^ we assessed whether reduced activation of the STAT3-cyclin D1 axis in tumor biopsies from HepG2-WT-HCMV-injected mice as compared with controls could also impact their tumorogenic potential. Therefore, we isolated the cells from the tumor tissue and determined their tumorogenic potential using a soft agar assay. We find the lowest number of colonies in soft agar seeded with the cells derived from HepG2-WT-HCMV tumor as compared with other groups (Figure 5). Altogether, lower Ki67 Ag expression and decreased STAT3 activation and cyclin D1 expression could explain the lower number of colonies in soft agar assay of tumors derived from wild-type HCMV-infected HepG2 cells and suggests an antiproliferative role of HCMV with decreased oncogenic properties *in vivo.*

We and others have shown that hepatocytes are permissible to the HCMV infection and that HepG2 cells can be infected with both laboratory and clinical HCMV strains with efficient viral entry, but limited viral growth.^29,45,46^ In addition, HCMV can induce lysis of primary human hepatocytes.^45^ Although we did not observe the lysis of HepG2 cells upon HCMV infection *in vitro* in a previous study,^29^ HCMV-induced apoptosis has been reported. Indeed, the infection of human cells with murine CMV activates the intrinsic apoptosis pathway mediated by caspase-9 in the human target cells.^34^ To further evaluate the role of apoptotic machinery in tumor delay, we assessed the expression of caspase-9 and caspase-3 proteins and transcripts in tumor samples using IHC, western blotting, and RT-PCR (Figure 6). We observed increased expression of caspase-3 and caspase-9 and their transcripts in tumor tissues from mice injected subcutaneously with HCMV-infected HepG2 cells, but not from the control groups. In addition, we also observed the increased expression of cytochrome c in the cytosolic enriched preparation of the tumor tissues isolated from HepG2-WT-HCMV group as compared with other groups (Figure 6). Our results indicate that the intrinsic apoptosis pathway is activated in tumors from mice injected subcutaneously with HCMV-infected HepG2 cells which could participate to the restriction of tumor growth in addition to the anti-proliferative effect observed as measured by decreased Ki67 antigen expression and the restricted transforming capacities as measured by limited activation of the STAT3-cyclin D1 axis and reduced numbers of colonies in soft agar.

We did not observe any significant activation of the intrinsic caspase pathway (as measured by caspase-3 and caspase-9 expression) in tumors of mice injected with HepG2 cells infected with UV-treated HCMV, indicating that active viral replication is required at least at some stages to restrict tumor formation *in vivo.* These findings are consistent with the previous published study^22^ where inactivated murine CMV virions failed to suppress the growth of lymphoma in murine model.

In agreement with Erlach *et al.*^22^ who reported lymphoma cell apoptosis in the liver induced by a distant mCMV infection, we detected caspase activation in lung tissues of xenografted mice injected with HepG2 cells infected with WT-HCMV (Figure 7), indicating that apoptosis induction was not totally restricted to the tumor tissue of mice injected subcutaneously with HCMV-infected HepG2 cells.

In a previous study, we infected HepG2 cells with HCMV strains AD169 (a laboratory strain) and HCMV-DB (a clinical isolate; accession number: KT959235)^46^ *in vitro*. We did not observe a highly productive infection of HCMV in HepG2 cells, indicating restricted and/or limited replication of HCMV.^29^ Additionally, we assessed the detection of the immediate early protein IE1 pp72, the early protein US28 and the late proteins pp65, and a 65-kD structural late antigen in HCMV-infected HepG2 cells using western blotting. We detected only the immediate early viral protein IE1, but neither the subsequently expressed US28 protein nor any of the late viral proteins.^29^ Our data indicate that most probably only part of the HCMV viral cycle occurs in infected HepG2 cells *in vitro* and that HCMV infection does not proceed beyond IE expression in these cells. Accordingly, we did not find HCMV major immediate early promoter DNA and IE1 antigen in tumor biopsies from mice injected subcutaneously with HCMV-infected HepG2 cells by PCR and IHC, respectively (Figure 3a,b). We also assessed the expression of IE1 and US28 gene transcripts in tumor biopsies from mice injected subcutaneously with HCMV-infected HepG2 cells and did not find US28 and IE1 transcripts at day 48 postinjection (Figure 3c). Therefore, although US28 has been reported to induce caspase-dependent apoptosis,^31^ it is unlikely that US28 protein is involved in tumor apoptosis observed in our murine model. Also due to the restricted HCMV growth in HepG2 cells *in vitro*, it is not surprising that we did not detect HCMV DNA neither in the tumors nor in lung and liver biopsies isolated from mice injected with HepG2 cells infected with wild-type HCMV.

We cannot rule out that other mechanisms than apoptosis and decreased proliferation are involved in restriction of tumor growth in our murine model. For instance, autophagy, mitotic catastrophe, paraptosis are few examples of alternate models of programmed cell death.^47^ Recently, Chaumorcel *et al.*^48^ reported that HCMV protein TRS1 negatively regulates autophagy in infected cells via its interaction with cellular autophagy protein Beclin 1 suggesting that HCMV genome is well equipped with antiautophagy machinery and thereby could modulate cell death.

In this study, we have used Balb/c nude mice which are deficient in T cells but have intact natural killer (NK) cell machinery. NK cells are the foremost cells of innate defense system with antitumoral and antiviral activities.^49^ In addition, HCMV secretome is very diverse, comprises of growth factors, extracellular matrix, extracellular matrix-modifying enzymes, cytokines, and chemokines.^50^ Mice and humans share several molecules involved in NK cells activation^51^ and NK cells can be activated in response to the cytokines and chemokines.^52^ Therefore, we raised the question whether HepG2 cells infected with wild-type HCMV implanted in Balb/c nude mice can trigger the induction of naive NK cells. We assessed the activation of the granzyme pathways, characteristic of cytotoxic cells activation, such as NK cells and cytotoxic T cells.^53^ We compared the expression levels of granzyme A (Gzm A) and B (Gzm B) among the three groups by reverse-transcriptase semiquantitative PCR (see Supplementary Figure S2a). We did not observe any significant variation in the expression of either Gzm A or Gzm B. We further assessed the gene expression of NKG2D protein which is expressed by cytotoxic cells, including NK cells, cytotoxic T cells, and macrophages.^54^ We did not detect significant differences in the levels of NKG2D gene expression among three groups (see Supplementary Figure S2b). Our results indicate that HCMV does not trigger NK antiviral response in our murine model as measured by NKG2D and granzyme gene expression. NKG2D receptor is selectively recognized by class of ligands such as MHC class I polypeptide-related sequences (MIC) A and B and UL16-binding proteins (ULBP1-6). In addition, HCMV has been shown to modulate the expression of these ligands *in vitro*.^55^ We have determined the expression of MIC-A/B and ULBP-1 using western blot. We did not find any significant difference in the level of these proteins among different groups (see Supplementary Figure S2c) (ULBP1; *P* = 0.43 (UI versus WT)); *P* = 0.15 (UV versus WT). MICA/B *P* = 0.58 (UI versus WT); *P* = 0.47 (UV versus WT). We are aware that these results could be different if murine CMV is employed in the study.

In conclusion, our results indicate that the intrinsic apoptosis pathway is activated in tumors from mice injected subcutaneously with HCMV-infected HepG2 cells. We also observed in tumors from mice injected subcutaneously with HCMV-infected HepG2 cells’ antiproliferative effects as measured by decreased Ki67 antigen expression and restricted transforming capacities as measured by limited activation of the STAT3-cyclin D1 axis and reduced numbers of colonies in soft agar. Induction of the intrinsic apoptotic pathway, limited cellular proliferation, and restricted transforming capacities could participate to the regression of human HCC observed in our murine model. Our study warrants the needs of development of humanized mice model for HCMV infection and might suggest HCMV as a future tool for virotherapy against HCC.

## Materials and Methods

### Reagents

Anti-cyclin D1, anti-Ki-67 Ag, anti-Stat3, anti-phosphoSTAT3, anti-HCMV-IE1 (pp72), anti-cytochrome c, anti-p53, anti-MDM2 antibodies were purchased from Santa Cruz Biotechnology (Santa Cruz, CA). Anti-β-actin antibody was purchased from Sigma-Aldrich (St. Louis, MO). Anti p21 was purchased from Cell signaling. Anti-caspase-3 (Cell Signaling Technology, Danvers, MA, clone 5A1E, 1:200) and anti-caspase-9 (Laboratory MBL, clone 5B4; 1:50), and anti-IE1-HCMV (Argene clone E13, 1:200, Varilhes, France) were used for IHC. Anti-MICA/B and ULBP1 antibodies were purchased from Abcam (Cambridge, UK).

### Cell culture

Human HCC cells (HepG2) (purchased from ATCC, Manassas, Virginia), were cultured in Dulbecco’s modified Eagle’s medium containing 10% fetal bovine serum (GE healthcare, Freiburg, Germany), penicillin (100 IU/ml) and streptomycin (100 µg/ml). MRC5 (human fibroblast cells) were cultured in EMEM supplemented with 10% fetal bovine serum, 1% nonessential amino acids (Sigma-Aldrich, St. Louis, MO), penicillin (100 IU/ml), and streptomycin (100 µg/ml).

### Virus propagation

HCMV laboratory strain AD169 was propagated in MRC5 and viral stocks were prepared as described earlier.^29^ AD169 is a highly passaged laboratory strain of HCMV originally isolated from the adenoids of a child.^56^ AD169 was tittered on MRC5 determined by standard plaque-forming assay.

### Animals

Six-week-old female Balb/c nude mice (Cby.Cg-Foxn1nu/J) purchased from Charles River Laboratories (L’Arbresle, France) were kept under strict pathogen-free conditions at the Central Animal facilities of University of Burgundy (Dijon, France). 

### Mice injection

HepG2 cells were either left uninfected or infected with UV-treated AD169 (1,200 µJ/cm^2^, 15 minutes) or with wild-type AD169 (MOI = 1). After 24 hours of infection, cells were harvested and cell viability was determined by trypan blue staining and cells were counted using haemocytometer. After harvesting and during injection, cells were maintained at 4 °C. Mice were divided into four groups. Group I: (*n* = 5) mice were injected with HepG2 infected with wild-type AD169 (HepG2-WT-HCMV); group II (*n* = 5) mice were injected with HepG2 infected with UV-treated AD169 (HepG2-UV-HCMV), group III (*n* = 5) were injected with uninfected HepG2 cells (HepG2-UI) and group IV (*n* = 2) comprises of mice with no injection. Balb/c-Nude mice were injected subcutaneously into the right flank with 5 × 10^6^ HepG2 cells (either uninfected or infected with wild-type HCMV or UV-inactivated HCMV) suspended in 100 µl of serum-free Dulbecco’s modified Eagle’s medium together with 100 µl of Matrigel at the day 0. Mice were checked twice in a week for tumor growth. Once tumor development was detectable by eye, tumor dimensions were measured using vernier caliper. Tumor volume was calculated using the formula: volume (cm^3^) = (*d* × *d* × *D*)/2 where *d* is the shortest diameter and *D* is the longest diameter.

### Animal killing

On day 48 postinjection, mice were killed according to the guidelines of the animal ethical committee. Organs (liver and lung) and tumor were retrieved from the mice. Half of the organs were preserved in ice-cold phosphate-buffered saline (PBS) for further analysis, and the rest half was preserved in formalin for IHC.

### Immunohistochemistry

Formalin-fixed organs and tumors retrieved from the mice were embedded in wax and sections (5 µm) were prepared using standard methods and stained with eosin and hematoxylin, as described previously.^57^ Sections were processed as described^57^ and were stained individually for hematoxylin and eosin staining, Ki67 antigen, caspase-3 (Cell signaling, clone 5A1E, 1:200), caspase-9 (Laboratory MBL, clone 5B4; 1:50) and IE1 of HCMV (Argene clone E13, 1:200) before observation by microscopy.^57^

### Polymerase chain reaction

The retrieved mice specimens (liver, lung, and tumor) were grinded in liquid nitrogen and DNA was isolated using QIAamp DNA mini kit (Qiagen, Valencia, CA) as per manufacture’s guidelines. Equal amount of DNA was analyzed by PCR using HCMV-major immediate early promoter-specific primers (see Supplementary Table S1). As equal loading control β-globin gene was amplified. For liver and lung mouse-specific β-globin primers were used and for tumor tissue human-specific β-globin primers were used, respectively. Sequences of the primers are given in Supplementary Table S1. Amplified product was electrophoresed in 2% agarose gel stained with Sybr green I nucleic acid stain.

### RT-PCR assay

Retrieved organs and tumors were grinded in liquid nitrogen and RNA was isolated using RNA mini kit (catalogue # 74104, Qiagen, Hilden, Germany) as per manufacturer’s instructions. RNA was quantitated and 2-µg RNA was reverse transcribed into cDNA using superscript III RT kit (Life technologies Carlsbad, CA) using oligo dT primers. Expression of viral transcripts (IE1, US28) and cellular genes (caspase-3, caspase-9, NKG2D, granzyme A, granzyme B, TNFR, TNFR2, Fas, and Fas-L) was determined using Sybr/Rox qPCR master mix (Qiagen, Germany) by real-time PCR relative Ct quantification method.^58^ Sequences of the primers used in the study are given in Supplementary Table S1 and source of primers are cited in Supplementary References.

### Western blot

Retrieved organs and tumors were grinded in liquid nitrogen and lysates were prepared in RIPA buffer as described previously.^29^ Expression of cyclin D1, caspase-3 and caspase-9, cytochrome c, STAT3, phosphoSTAT-3, Ki67 Ag, p53, p21, MDM2, ULBP1 and MICA/B was determined by western blotting as previously described.^29^ For cytochrome c assay, cytosolic enriched extracts were prepared from the fresh tumor tissue as described previously.^59^ As a loading control β-actin was included in the study.

### Soft agar assay

Single cells were isolated from tumor tissue of mice groups (uninfected, UV-HCMV infected and wild-type HCMV-infected HepG2) as described.^60^ Five thousand cells were assessed for colony formation in soft agar assay using Cell Biolabs Cytosolic Cell Transformation Assay kit (Colorimetric assay, CB135; Cell Biolabs, San Diego, CA) as per manufacturer’s instructions. Post 8 days of inoculation, colonies were observed under an Olympus microscope. In addition, cell proliferation was determined using MTT assay as previously described.^29^

### Agarose-based apoptotic DNA ladder assay

Apoptotic DNA ladder assay was performed as described earlier^61^ with few modifications. Tumor tissue was grinded in liquid nitrogen and genomic DNA was isolated using Qiamp DNA blood mini kit (Qiagen, cat # 51106). Purified DNA was quantitated using spectrophotometer and analyzed on 2% agarose gel and visualized by Sybr Green I nucleic acid gel stain (Lonza, cat # 50512).

### Statistical analysis

The reported values are the mean values and SD or SEMs of independent experiments. Statistical analysis was performed using the Student’s *t*-test and differences were considered significant at a *P* value < 0.05. Microsoft Excel was used to construct the plots.

Animals care and related procedures were performed according to the guidelines of ethical committee for Animal experimentation of the University of Burgundy.

## Notes

The first two authors contributed equally to this work.

## Notes

Supplementary Figures and Table References
