# Supplementary material for: Tumor control by human cytomegalovirus in a murine model of hepatocellular carcinoma
Source: Mol Ther Oncolytics. 2016 Apr 27;3:16012–. doi: 10.1038/mto.2016.12 (PMC5008266; doi:10.1038/mto.2016.12)
Supplement: Supplementary Figures and Table References [file mto201612-s1.zip › supplementary table and references.pdf]

Suppl. Table 1: Sequence of the primers used in the study.

Primer	Sequence	Reference
MIEP-F	5′-AAC TCC CAT TGA CGT CAA T-3′	
MIEP-R	5′-TGG GAC TTT CCT ACT TGG-3′	
Granzyme A F	5′-GAC TGC TGC CCA CTG TAA CG-3′	1
Granzyme A R	5′-TCA ATA TCT GTT GTT CTG GCT CCT TA-3′	1
Granzyme B F	5′-TGT CTC TGG CCT CCA GGA CAA-3′	1
Granzyme B R	5′-CTC AGG CTG CTG ATC CTT GAT CGA-3′	1
IE1 F	5′-CTC TGT CCT CAG TAA TTG TGG CTG-3′	2
IE1 R	5′-GCA ACT TCC TCT ATC TCA GAC ACT G-3′	2
US28 F	5′-AGC GTG CCG TGT ACG TTA C-3′	2
US28 R	5′-ATA AAG ACA AGC ACG ACC-3′	2
NKG2D R	5′-GTG TTA AGG GTG AAT CGA ATT GC-3′	1
NKG2D F	5′-TAT CAT AAG AGG AGG ATA CCC TAT AGA AA-3′	1
b-globin F-h	5′-TCC-CCT-CCT-ACC-CCT-ACT-TTC-TA-3′	2
b-globin R-h	5′-TGC-CTG-GAC-TAA-TCT-GCA-AGA-G-3′	2
b-globin F-m	5′-ATG-GTG-CAC-CTG-ACT-GAT-GCT-GAG-AA-3	
b-globin R-m	5′-ACC-AAC-TTC-ATC-GGA-GTT-CAC-C-3	
Caspase 3 F	5′-TGG TTC ATC CAG TCG CTT TG-3′	3
Caspase 3 R	5′-CAT TCT GTT GCC ACC TTT CG-3′	3
Caspase 9 F	5′-CGA ACT AAC AGG CAA GCA GC-3′	3
Caspase 9R	5′-ACC TCA CCA AAT CCT CCA GAA C-3	3
Caspase 8 F	5′-GCC TCC CTC AAG TTC CT-3′	3
Caspase 8 R	5′-CCT GGA GTC TCT GGA ATA ACA-3′	3
Fas F	5′-TGC AGA AGA TGT AGA TTG TGT GAT GA-3′	4
Fas R	5′-GGG TCC GGG TGC AGT TTA TT-3′	4
Fas-L F	5′-CCA TGT GAA GAG GGA GAA GC-3′	5
Fas-L R	5′-AAG ACA GTC CCC CTT GAG GT-3′	5
TNFR1 F	5′-CGC TAC CAA CGG TGG AAG TC-3′	6
TNFR1 R	5′-CAA GCT CCC CCT CTT TTT CAG-3′	6
TNFR2 F	5′-CAA GCC AGC TCC ACA ATG G-3′	6
TNFR2 R	5′-TGA CCG AAA GGC ACA TTC CT-3′	6
