# Supplementary figures and images for: Tumor control by human cytomegalovirus in a murine model of hepatocellular carcinoma
Source: Mol Ther Oncolytics. 2016 Apr 27;3:16012–. doi: 10.1038/mto.2016.12 (PMC5008266; doi:10.1038/mto.2016.12)

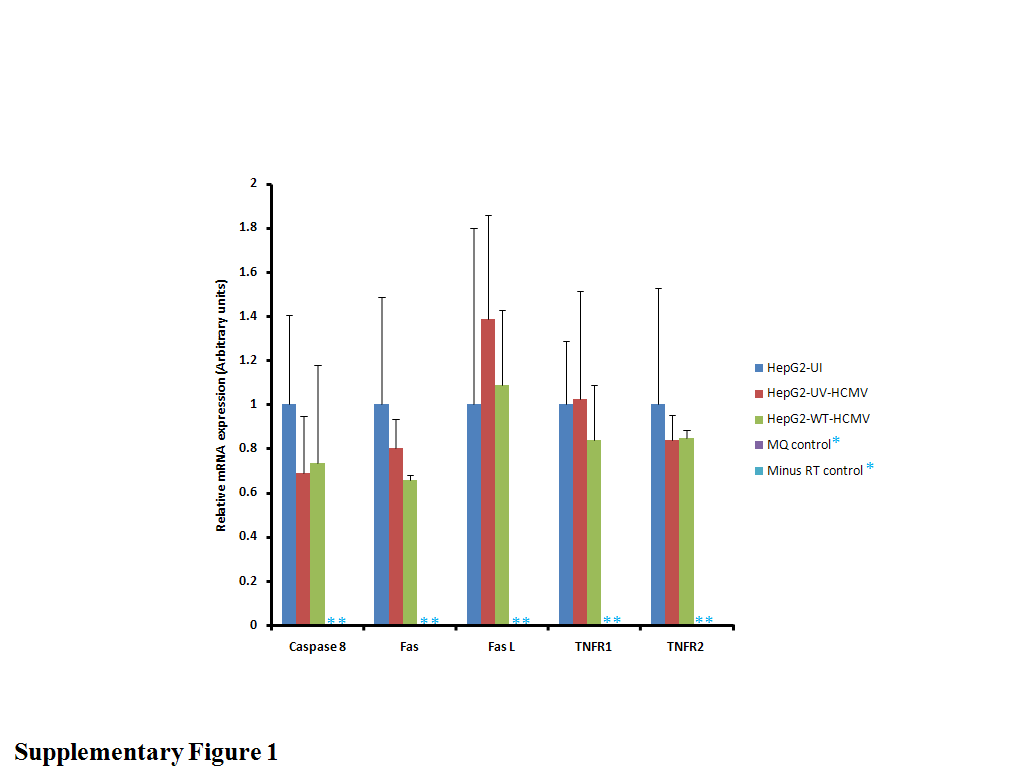

Supplement: Supplementary Figures and Table References [file mto201612-s1.zip › mto-00077-s01.tif]

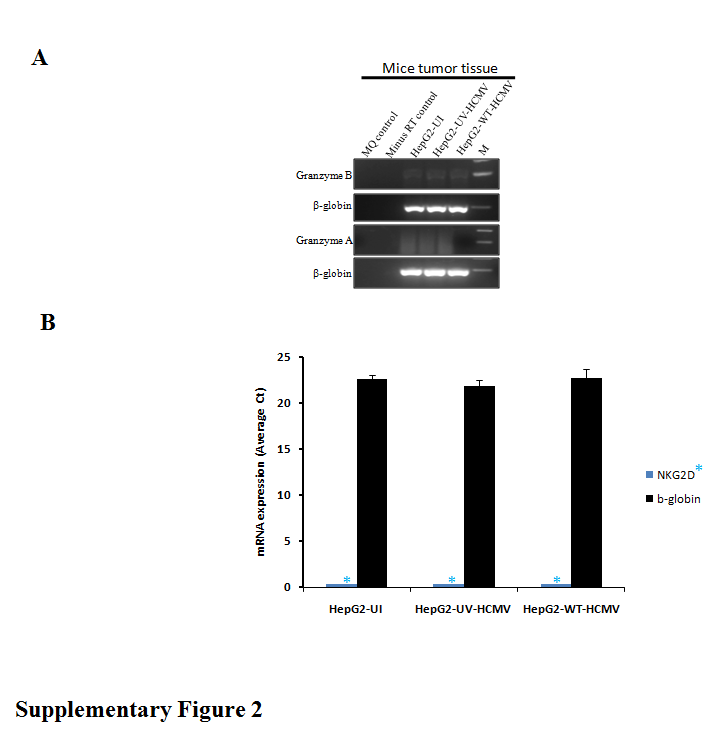

Supplement: Supplementary Figures and Table References [file mto201612-s1.zip › mto-00077-s02.tif]

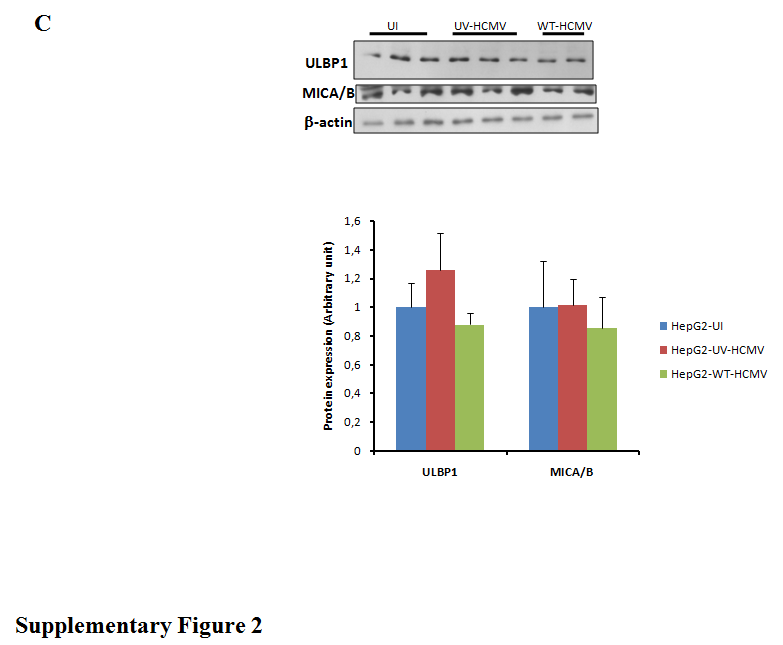

Supplement: Supplementary Figures and Table References [file mto201612-s1.zip › mto-00077-s03.tif]
